# Isokinetic Muscle Strengthening of the Lower Limbs in People with Stroke: A Systematic Review of Randomized Controlled Trials

**DOI:** 10.3390/jcm14072215

**Published:** 2025-03-25

**Authors:** Elsa Alejandra Aguirre-Villanueva, Roberto Cano-de-la-Cuerda, Sofía Laguarta-Val

**Affiliations:** Department of Physical Therapy, Occupational Therapy, Physical Medicine and Rehabilitation, Faculty of Health Sciences, Rey Juan Carlos University, 28922 Alcorcón, Spain; ea.aguirre.2023@alumnos.urjc.es

**Keywords:** isokinetic muscle strengthening, isokinetic training, stroke, gait, lower limb

## Abstract

**Introduction:** Muscle weakness is one of the main consequences following a stroke, leading to significant alterations in gait and other daily activities. Isokinetic muscle strengthening of the lower limb is considered to be an effective complement to conventional treatment to improve these functional limitations. The objective of this systematic review was to analyze isokinetic lower limb strengthening protocols and their effects on muscle strength, gait, and mobility in post-stroke patients compared to conventional physiotherapy or other strengthening methods. **Methods:** A systematic review of randomized controlled trials (RCTs) from the last 10 years was conducted. Studies analyzing the effects of isokinetic lower limb muscle strengthening compared to conventional physiotherapy or other strengthening methods on muscle strength, gait parameters, and mobility in post-stroke adults were selected. The quality and risk of bias of the studies were evaluated using the PEDro scale, the Oxford Level of Evidence scale, and the Cochrane Review Manager tool. **Results:** Six studies met the eligibility criteria. For knee–ankle strength, gait speed, and mobility, isokinetic strengthening demonstrated significant improvements compared to conventional physical therapy. When compared to other strengthening methods, isokinetic training was more effective for hip–knee strength and mobility, while closed kinetic chain isokinetic strengthening showed greater benefits for gait speed. Additionally, in the early post-stroke phase, isokinetic training showed greater improvements, while the chronic phase demonstrated more variable results. The methodological quality of the studies was acceptable–good. **Conclusions:** Isokinetic muscle strengthening significantly improves muscle strength, gait speed, and mobility in post-stroke adults compared to conventional physical therapy, offering additional benefits over other strengthening methods. Further research is needed to evaluate its long-term effectiveness, optimize protocols, and explore the impact of treatment timing.

## 1. Introduction

Strokes are caused by the obstruction of or reduction in blood flow in a certain region of the brain, producing dysfunction in the affected area [1,2].

In Spain, strokes are the leading cause of death in women and the second in men, making it the main cause of disability and dependence. It is estimated that around 50% of survivors develop some degree of disability, and most will experience long-term effects that reduce their independence and quality of life [3].

This condition affects all domains of the International Classification of Functioning, Disability, and Health (ICF). Muscle weakness is one of the most common consequences, limiting mobility, walking, and daily activities (ADLs) [4]. It affects 89% of patients in the first week after a stroke, 72% within the first month, and 61% after six months [5].

Additionally, muscle atrophy due to lower limb paresis is not limited to the affected side. It occurs bilaterally and is directly linked to reduced walking speed, leading to lower physical performance [6]. Gait plays a crucial role in post-stroke recovery within the ICF framework. Muscle function recovery in the lower limb is essential for reintegration into daily life. Progressive resistance training has been shown to improve knee flexor and extensor strength, thus promoting reintegration [4].

Studies suggest that half of the patients who receive rehabilitation within the first six months after a stroke recover full motor function, and 60–70% regain independence in ADLs. However, between 50% and 75% are unable to return to work [3].

The concept of isokinesis refers to the ability to maintain a constant speed of muscle contraction during movement, allowing for a more accurate measurement of strength and performance. In rehabilitation, isokinetic equipment is widely used for muscle recovery, providing safe and controlled intensity training [7]. This equipment includes an isokinetic dynamometer, which measures peak force moments through torque during dynamic eccentric and concentric contractions, adjusting to a specific angular velocity [5].

Isokinetic equipment has been used in rehabilitation for the evaluation and treatment of patients with traumatological or sports pathologies. Trends in neurorehabilitation are changing, and muscle strengthening techniques present a high incorporation into clinical protocols. Muscle strengthening is crucial in post-stroke rehabilitation. Since the weakness affects not only the paretic side but both lower limbs, a critical systematic review, such as the one we have outlined, is essential.

Clinical trials in this population are scarce, and the samples are very small. Therefore, our review will be useful for clinicians, like our group, who have experience in the treatment of post-stroke patients and in the use of isokinetics, so that they can use the tool safely and with guaranteed efficacy.

Before starting the test or training session, the patient is seated and securely positioned with straps to minimize compensatory movements. They are instructed to push during the concentric phase and resist against the machine during the eccentric phase. Additionally, they are guided to move at the set speed within the range of motion they are able to perform, ensuring consistency in the assessment or strengthening program. This approach allows for the accurate strength evaluation and tracking of functional recovery [7].

Although previous studies have demonstrated the benefits of isokinetics in patient recovery, inconsistencies in equipment and treatment methods prevent comparison between rehabilitation protocols [5,7]. Additionally, differences in access to rehabilitation services, such as delays in treatment initiation, significantly affect recovery outcomes. This highlights the need for optimized and timely interventions [8].

To overcome this, this review gathers the available evidence, providing insights into how isokinetic strengthening compares across different protocols and methods. Consistent evidence supports the benefits of isokinetic muscle strengthening in preventing atrophy and promoting patient recovery following prolonged inactivity [7] and cerebrovascular accidents. However, its application requires further exploration.

This systematic review intends to identify, compare, and analyze isokinetic lower limb strengthening protocols for post-stroke adults. Key characteristics, dosage, and main outcomes will be examined, focusing on their effects on muscle strength, gait parameters, and mobility. Additionally, these protocols will be compared to conventional physiotherapy and other strengthening methods to provide a comprehensive overview of rehabilitation options.

Furthermore, this review intends to serve as a structured and practical resource for healthcare professionals and researchers, therefore helping them to implement effective treatment strategies based on patient needs, treatment duration, and the availability of specific equipment in different clinical settings.

## 2. Methods

### 2.1. Design

This systematic review was conducted according to the guidelines of the PRISMA 2020 statement for the development and publication of systematic reviews [9]. It began with the development of the PICO (population, intervention, comparison, and outcome) question: How do isokinetic lower limb strengthening protocols compare in terms of key characteristics, dosage, and effects on muscle strength, gait parameters, and mobility, relative to conventional physiotherapy or other strengthening methods?

The objective of this review was to analyze isokinetic lower limb strengthening protocols and their effects on muscle strength, gait, and mobility in post-stroke patients compared to conventional physiotherapy or other strengthening methods.

The review protocol was not registered.

### 2.2. Search Strategy and Selection of Studies

Bibliographic searches were carried out using PEDro, PubMed, Scopus, Cochrane, Medline, Web of Science, and Dialnet. Other sources such as Google Scholar were consulted, and relevant references of the same studies were reviewed.

The keywords searched were isokinetic strength, isokinetic training, stroke, and lower limb, using Boolean operators and specific search filters (isokinetic strength OR isokinetic training AND stroke AND lower limb) (Table 1).

The corresponding databases were searched independently, analyzing the titles and abstracts to determine whether or not they met the eligibility criteria.

### 2.3. Eligibility Criteria

#### 2.3.1. Types of Studies

Randomized controlled clinical trials published in the last 10 years were included, with 2014 being the last year considered and without language restrictions. Studies that used isokinetic equipment as a method of evaluation and not as a treatment were excluded.

#### 2.3.2. Types of Participants

Participants had to be adults with post-stroke sequels at any stage of the post-stroke period, regardless of age or sex.

#### 2.3.3. Types of Interventions

This review included all the available studies on isokinetic lower limb strengthening for post-stroke patients within the specified timeframe. The intervention had to involve isokinetic strengthening, using isokinetic dynamometry for evaluation, regardless of the equipment used (i.e., Biodex System, CSMI Humac/Norm) or joints involved (hip, knee, and/or ankle). Both concentric and eccentric contractions were analyzed, as well as dosage variables (speed, repetitions, sets, and rest) and treatment duration (weekly and total).

The studies in this review followed different isokinetic protocols, with variations in angular velocity, frequency, and session duration. Only studies that clearly specified these parameters were included. Protocol variability was acknowledged and considered during the analysis of the results. The purpose was to compare different isokinetic strengthening methods and their effects on strength, gait, and mobility.

#### 2.3.4. Types of Outcome Measures

Force measurement was performed with the same isokinetic equipment, using peak torque (PT), expressed in Newton meter (Nm), and all studies included at least one gait or mobility assessment.

Gait parameters could be assessed using spatiotemporal scales or specialized gait analysis devices. Mobility was assessed by scales measuring motor function or lower limb functionality.

### 2.4. Data Extraction

The following information was collected from each study: number of participants and main characteristics (age and sex), length of intervention (months), design (group division), isokinetic equipment, selected joints, movements to be performed, and previous conditioning and active recovery (if applicable).

Also, the type of contraction used, treatment dosage (repetitions), weekly and total time, evaluation methods, results (if there were statistical or clinically significant differences between the groups), patients who did not complete the study, and conclusions were obtained.

### 2.5. Assessment of the Quality and Risk of Bias of the Individual Studies

The methodological quality of the included studies was assessed using the PEDro scale, which was extracted directly from the evidence-based physical therapy database [10]. All included studies were evaluated, and the scores were corroborated using the aforementioned scale. The scoring criteria for assessing the quality of studies using this scale can be excellent (9, 10), good (6–8), acceptable (4, 5), and poor (≤3) [11]. A higher score indicates better methodological quality, with emphasis on factors such as randomization, blinding, allocation concealment, and statistical analysis of key outcomes.

The Oxford scale was used to evaluate the level of evidence and assign a grade of recommendation [12]. The Cochrane Review Manager program (https://training.cochrane.org/online-learning/core-software, accessed on 3 March 2024) was used to assess the validity of the articles included and to determine the risk of bias in the results. This program is a software that provides a description and assessment for each randomized controlled trial [13]. Studies were evaluated by answering a question that indicates a low, high, or uncertain risk of bias, reflecting the lack of information or uncertainty.

## 3. Results

The literature search identified 69 records after removing duplicates (*n* = 31). Title and abstract screening excluded 23 articles. Therefore, 46 full-text articles were analyzed according to the inclusion and exclusion criteria.

**Figure 1:**
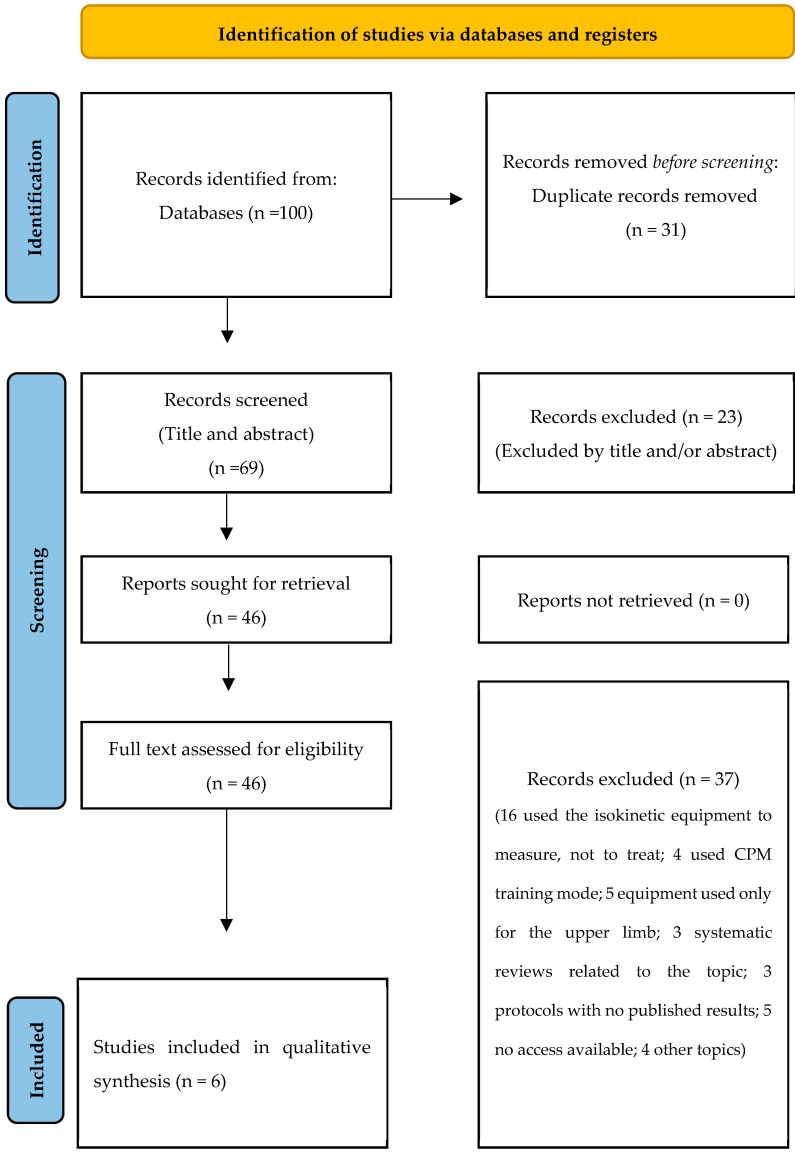
Flowchart.

Of these, 37 studies were excluded for certain reasons, such as using the equipment only for measurement, applying it only to the upper limb, using different training methods, or having no published results [Figure 1]. Finally, six studies with 251 participants met the eligibility criteria and were included in the analysis (Table 2). All studies measured strength by peak torque, five analyzed at least one gait parameter [14,15,16,17,18], and two examined mobility [14,19].

Of the included studies, five were written in English and one in Chinese [19]. For the Chinese study, DeepL Translate was used, and both the summary and result tables were available in English.

### 3.1. Characteristics of the Participants

In five studies, the participants in the isokinetic group had an average age of 54.5 ± 6.27 years (range: 47.93–64.7), while the control group had an average age of 56.76 ± 5.84 years (range: 53–67.1). The total number of participants was 105 men and 59 women. One study did not specify the gender, only stating that patients were aged between 60 and 75 years [19].

The time from stroke to treatment initiation for participants in the isokinetic group was 17.18 ± 24.06 months (range: 3–59), while in the control group, it was 17.02 ± 22.70 months (range: 3–57). One study only mentioned that its patients were at least 2 months post-stroke [19].

Out of the six studies, five reported no participant loss, while one had significant losses, exceeding 20% of its participants [15].

### 3.2. Characteristics of the Interventions

All studies were randomized controlled trials, divided into intervention and control groups (Table 2). Two studies compared isokinetic muscle strengthening with conventional physiotherapy sessions [14,17], while four studies compared isokinetic training with other forms of strengthening or exercise, including conventional physiotherapy [15,16,18,19].

Regarding the joints involved, three studies focused on strengthening only the knee musculature [15,18,19], two studies strengthened both the knee and ankle [14,17], and one study strengthened the hip, knee, and ankle musculature [16].

The concentric muscle contraction was employed in all studies, except for one that combined concentric and eccentric contraction [15].

Two studies performed bilateral training [14,15], while four focused only on the affected side [16,17,18,19].

The training frequency was 4 ± 1.09 sessions per week (range: 3–5), and the treatment lasted 5 ± 1.83 weeks (range: 3–8), with a total of 19 ± 2.99 sessions per intervention (range: 15–24) (Table 3).

Four studies used the Biodex System [13,14,16,17] and two Humac/Norm [15,18] as the method of evaluation and treatment. Isokinetic strengthening protocols were divided into two categories: conditioning and recovery (if mentioned) and training dosage. Three studies reported conditioning before isokinetic training [14,18,19], and two studies mentioned including a recovery phase after using the equipment [18,19].

For training dosage, repetitions ranged from 5 to 10, with angular velocities between 60°/s and 180°/s. On average per session, about 15 hip flexions and extensions, 43.33 ± 36 knee flexions and extensions, and 23.33 ± 7.63 dorsal and plantar ankle flexions were performed. The rest periods between series were 10 s [14,17,18], 15 s [19], with 1 min between different angular speeds [19], or 2 min between series [18] (Table 4).

### 3.3. Outcome Measures

The assessed variables included muscle strength, measured by peak torque (Nm), as well as gait and mobility parameters, evaluated using different scales and systems (Table 4).

For strength, velocities that ranged from 60°/s to 180°/s were used, with 60°/s being used in five studies [14,15,16,18,19]. For gait, five studies analyzed it using different scales and/or parameters [14,15,16,17,18]. Three evaluated speed [14,17,18], three used the Timed Up and Go Test [14,15,17], and two used the Six-Minute Walk Test [14,16].

Additionally, one study used the Qualisys Motion Capture System to measure gait speed, cycle time, and lower limb support [17], and another used the GAITRite mat to record speed, step length, and swing phase duration [18]. In relation to lower limb mobility, one study used the Fugl-Meyer lower extremity scale [19] and another used the Rivermead Mobility Index along with the Functional Independent Measure [14].

### 3.4. Results of the Individual Studies: Effects of the Interventions

The results of the interventions, divided by groups, are presented below. It should be noted that both the intervention and control groups received conventional physiotherapy. However, the intervention groups also performed isokinetic muscle strengthening, while the control groups performed other types of strengthening or exercise, according to each study (Table 2 and Table 4).

### 3.5. Comparison Between Isokinetic Muscle Strengthening and Conventional Physical Therapy

Two studies (*n* = 80) reported statistically significant results in favor of isokinetic strengthening, increasing the PT of the knee and ankle muscles at angular velocities of 30°/s to 180°/s. Improvements were shown in terms of mobility and gait speed, measured by tests such as the 10-Meter Walk Test, Timed Up and Go Test, Qualisys Motion Capture System, Rivermead Mobility Index, and others. Benefits in terms of balance and quality of life were also observed [14,17].

### 3.6. Comparison Between Isokinetic Muscle Strengthening and Other Strengthening Methods

One study (*n* = 31) compared isokinetic training with isotonic training, finding significant results in bilateral knee flexion and extension PT in the isokinetic group. The isotonic group showed improvements only in the healthy lower limb. The isokinetic group had significant results for knee flexion PT on the affected side at 60°/s and 120°/s.

Both groups showed improvements in the Timed Up and Go Test and the physical functioning domain of the SF-36. However, only the isokinetic group obtained significant results in the physical subdomain and the total score, with no significant differences between groups in both tests [15].

Another study (*n* = 30) compared isokinetic muscle strengthening with functional strengthening, showing statistically significant effects on hip and knee PT and the Six-Minute Walk Test in both groups. The isokinetic group showed greater benefits. For ankle PT and step length, both groups showed significant improvements, with no differences between them [16].

One study (*n* = 30) compared isokinetic training in open kinetic chain versus closed kinetic chain, with significant results in both groups for knee and ankle PT. There were no differences between the groups. For the gait parameters measured by the GAITRite mat, both interventions had significant effects, with the closed kinetic chain group showing better results [18].

One study (*n* = 80) compared the combination of conventional physical therapy, aerobic exercise, and isokinetic strengthening with conventional physical therapy and aerobic exercise. Both groups obtained statistically significant results, but the group with isokinetic strengthening showed greater benefits in terms of knee PT, lower limb Fugl-Meyer assessment, and cardiovascular measures, suggesting that adding isokinetic strengthening provides additional advantages over conventional rehabilitation [19].

### 3.7. Comparison of Early (<6 Months) and Chronic (>6 Months) Post-Stroke Treatment Effects

**Figure 2:**
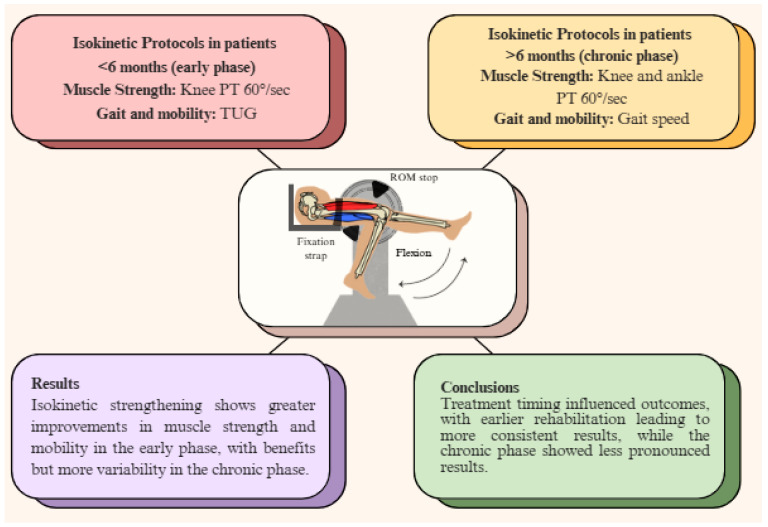
Common outcome measures and results by time post-stroke (before or after 6 months).

Studies were classified based on the timing of treatment: early (<6 months) and chronic (>6 months) post-stroke. In the early phase, patients receiving isokinetic strengthening demonstrated significant improvements in muscle strength and mobility (e.g., 6MWT and TUG) compared to the conventional physiotherapy group. In the chronic phase, improvements in muscle strength, gait speed, and mobility were still observed, but these were less pronounced. Despite this, isokinetic strengthening continued to provide benefits, although the results were more variable in this phase (Table 5, Figure 2).

### 3.8. Effect Size and Statistical Measures

Five studies did not report effect size, while only one study [18] provided clinical significance for gait speed. This study showed an effect size of 0.10 m/s for open kinetic chain (OKC) and 0.14 m/s for closed kinetic chain (CKC) exercises. The minimum clinically important difference for gait velocity was set at 0.10–0.20 m/s [18].

The remaining studies mainly reported statistical measures such as *p*-values, *t*-values, change levels, or percentages of improvement (Table 6).

### 3.9. Assessment of the Quality and Risk of Bias of Individual Studies

The methodological quality of the included studies was assessed using the PEDro scale, with an average score of 6 out of 10, indicating an overall quality ranging from acceptable to good. Two studies presented acceptable score [15,16], and the remaining four achieved good quality, with 6 or 7 points [14,17,18,19] (Table 7). Studies with higher scores were considered to have lower risk of bias, particularly in terms of randomization, blinding, and outcome reporting.

The level of evidence and grade of recommendation were analyzed with the Oxford scale, and all the studies had evidence of type 1b or 2b and grade of recommendation A or B (Table 8).

**Figure 3:**
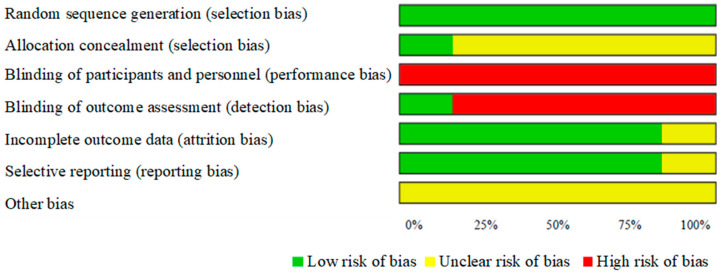
Risk of bias: reviewer judgment of the risk of bias represented as percentages of all included studies.

**Figure 4:**
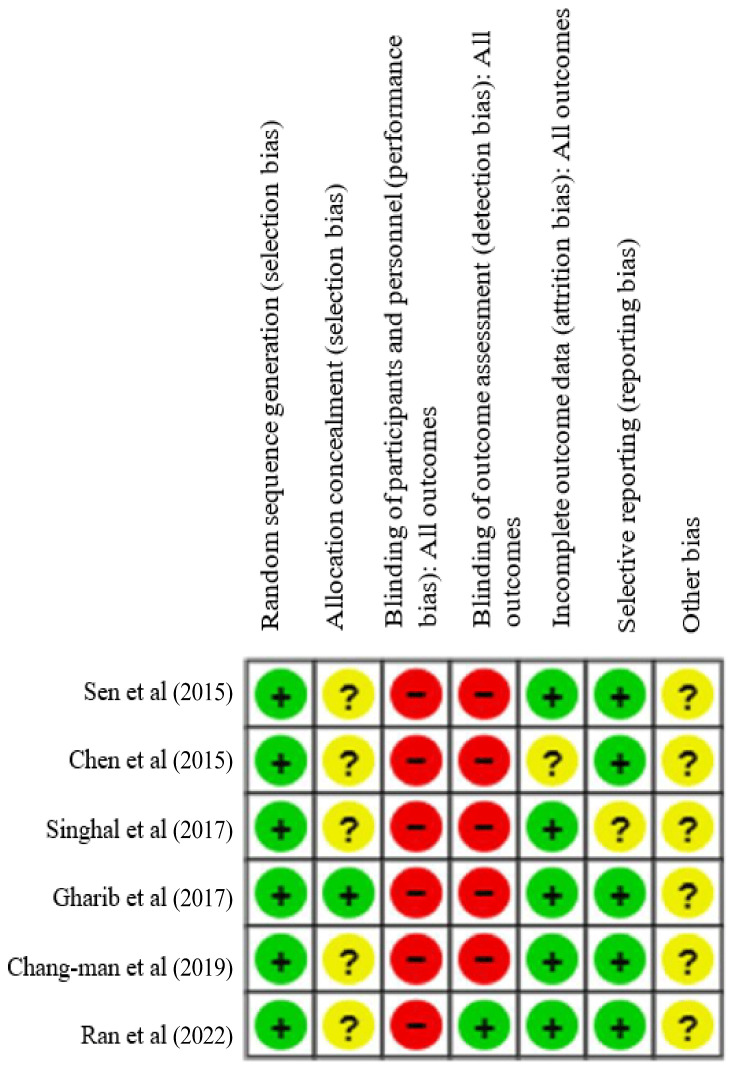
Summary of risk of bias: reviewer judgment of the risk of bias for each included study [14,15,16,17,18,19].

The risk of bias assessment for the included studies, both overall and individually, showed that all studies (100%) implemented proper randomization. However, only 16.6% clearly reported allocation concealment and assessor blinding, and none applied blinding to therapists or participants. On the other hand, 83.3% provided complete outcome data and demonstrated good outcome management [Figure 3 and Figure 4].

## 4. Discussion

This review demonstrates that isokinetic training, whether bilateral or focused on the affected side, significantly improves muscle strength, gait speed, and mobility when compared to conventional physiotherapy. It also contributes to better balance and quality of life in post-stroke patients [14,15]. This is because rehabilitation robots promote muscle activation, coordination, and neural plasticity through repetitive, specific movements and exercises. In comparison to traditional rehabilitation therapies, lower-limb rehabilitation robots provide more effective neural stimulation, facilitating the recovery of lower-limb function [20].

Supporting these findings, two systematic reviews with meta-analyses from 2019 provide further evidence. One analyzed 13 studies (*n* = 347) and reported significant improvements in muscle strength, mobility, and gait, although it highlighted the need for more randomized controlled trials [21]. The second review (*n* = 154) found that robotic isokinetic training benefits patients with hemiplegia, improving lower limb motor function, gait speed, stair climbing, balance, functional independence, and quality of life [22]. These results align with other evidence suggesting that incorporating isokinetic muscle strengthening into conventional physiotherapy and aerobic exercise further improves strength, lower limb function, and cardiopulmonary fitness [19].

However, when comparing isokinetic strengthening with other methods, results vary depending on the analyzed variable. Compared to isotonic strengthening, the isokinetic group shows notable improvement, but no statistically significant differences, except for increased knee flexion strength on the affected side [15].

These results might be attributed to the moderate volume and intensity in both groups; the isokinetic group performed 15 concentric and 15 eccentric contractions, while the isotonic group completed 30 repetitions at 60% of maximum strength, which might not be sufficient to create significant differences. Additionally, the small sample size, with losses exceeding 20%, and the use of the TUG Test and SF-36 (although valuable outcome measures), may not be sensitive enough to detect differences in this context.

In contrast, compared to functional strengthening, isokinetic training leads to better hip and knee peak torque (PT) and improved 6-Minute Walk Test results. However, no significant differences were found in ankle PT or step length between groups [16]. A possible explanation is that only 15 repetitions per joint were performed at a single speed (60°/s), which may have limited improvements in ankle PT and step length. This idea is supported by studies showing better strength gains when training at different angular velocities [23], as muscle responses change depending on the speed of movement during contractions [24].

When comparing open kinetic chain (OKC) and closed kinetic chain (CKC) isokinetic training, both methods significantly increase knee and ankle PT, with no major differences between them. However, for gait velocity and spatial symmetry, results favor CKC training [18]. These findings could be explained by the use of a “leg press” accessory in the CKC group. This device prevents knee isolation, leading to greater lower limb muscle activation and improved gait performance compared to OKC training. These results align with a systematic review on post-stroke muscle strengthening, which found that studies using “leg press” equipment had a positive impact on strength and gait [25].

The differences observed, though mostly positive, could be due to the methodological heterogeneity of the included studies. This variability may be influenced by factors such as the time post-stroke when participants received treatment, age, group distribution, joints selected, type of contraction used, and, particularly, the variety of outcome measures and training dosages applied.

The findings from this study emphasizes the importance of treatment timing in stroke rehabilitation. Early interventions (<6 months) lead to more consistent and significant improvements in muscle strength and mobility compared to conventional physiotherapy, supporting previous findings [15], and can significantly improve the self-care abilities, daily activities, and neurological functions of ischemic stroke patients [26].

In the chronic phase (>6 months), isokinetic strengthening still showed benefits, but with more variability. Stroke remains a major cause of long-term disability, often impairing motor function, balance, and quality of life [27], which may contribute to this variability. Differences in stroke duration, severity, and rehabilitation protocols, along with the heterogeneity of studies, complicate drawing definitive conclusions. This suggests that the chronic phase may present unique challenges that affect outcome consistency.

### 4.1. Limitations

This review has several limitations. One of them is the criterion of including only studies from the last ten years, which may have reduced the number of studies analyzed. Some articles from this period were not available in full text. Additionally, the use of isokinetic equipment was limited to strengthening, excluding studies that used the CPM mode, which involves passive mobilizations by the same equipment.

The methodological quality of the studies ranged from acceptable to good, according to the PEDro scale. However, the results should be interpreted with caution, as none of the studies used blinding for patients and therapists. Only one study blinded the evaluators, and most studies did not clearly describe the randomization concealment, which could lead to significant biases.

Also, the lack of homogeneity in participant characteristics, variability in training dosages, and small sample sizes in most studies limit the generalizability of the findings. Isokinetic training is widely used for muscle strengthening in athletes and healthy individuals, as well as for studying the effects of different velocities and types of muscle contractions.

Since this review focuses on post-stroke patients and the application of isokinetic training as both an evaluation and treatment method, the available information on its use in neurological patients is limited. Finally, although isokinetic equipment is a valuable tool for rehabilitation, it is not available in all clinical settings due to its high cost and the need for specialized personnel. It requires direct supervision by healthcare professionals, which increases treatment costs and limits the number of patients who can be treated daily. As a result, it is, in some cases, a less accessible resource for patients [8].

### 4.2. Clinical Implications of the Review and Future Research Lines

Muscle strengthening is crucial in rehabilitation following a stroke. Since weakness affects not only the paretic side but also both lower limbs, it is essential to include bilateral training to maximize the benefits of treatment [21]. When available, the use of isokinetic equipment in this context has shown more significant results compared to conventional physiotherapy, improving both physical and functional outcomes in patients.

Future research should explore the potential of combining concentric and eccentric isokinetic training to further optimize clinical outcomes. Recent studies suggest that eccentric training may be preferable in certain clinical contexts due to its positive effects on muscle strength [24,28]. It is also important to implement long-term follow-ups to assess the durability of treatment effects, especially considering that access to this equipment may be limited for some patients.

Regarding research quality, it is essential to improve the reporting and design of future studies. Increasing the number of randomized controlled trials and conducting systematic reviews with meta-analyses will provide stronger evidence on the effects of isokinetic strengthening in post-stroke recovery.

The use of robust and standardized outcome measures will be crucial to accurately and comparatively assess the benefits of these interventions. This will offer clearer guidance for clinical practices, improve patient outcomes, and validate the complementary use of this technique in post-stroke neurorehabilitation.

Future research should focus on using larger sample sizes to enhance the reliability of the results. Multi-center trials are needed to improve generalizability, and long-term follow-up studies will be crucial for evaluating the sustainability of treatment effects. Given the high cost and limited availability of isokinetic equipment, exploring cost-effective alternatives that may offer similar benefits for stroke rehabilitation is essential.

Furthermore, future studies should include effect size calculations, such as Cohen’s D, along with statistical significance measures. Relying solely on *p*-values does not provide insights into the clinical relevance or practical impact of interventions. This would help to better understand the true magnitude of improvements, particularly in terms of muscle strength, gait speed, and mobility.

## 5. Conclusions

This review analyzed the effects of isokinetic lower limb strengthening on muscle strength, gait, and mobility in post-stroke patients compared to conventional physiotherapy or other strengthening methods.

Improvements in muscle strength and mobility were observed, with gait speed being the only measure that showed a significant change. Treatment timing influenced the outcomes, with earlier rehabilitation (<6 months) leading to more consistent results, while the chronic phase (>6 months) showed more variability.

Although isokinetic protocols may offer potential benefits, further research is needed to understand their long-term effects and optimal implementation. Limitations such as small sample sizes and protocol variability emphasize the need for standardized protocols and larger trials to evaluate their effectiveness in post-stroke rehabilitation.

## Figures and Tables

**Table 1:** Search filters in databases.

Dialnet	Type of article: journal article Publication years: 2014–2024
PEDro	Publication years: 2014–2024
PubMed	Article accessibility: full text Publication date: last 10 years
SCOPUS	Document type: clinical trial Publication years: 2014–2023
Cochrane	Publication years: 2014–2024
MEDLINE	Publication years: 2014–2023
Web of Science	Publication years: 2014–2024

**Table 2:** Key characteristics: data about participants, interventions, joints, movements, and type of contraction used.

	Participants	Intervention Group	Control Group	Joints and Movements
Sen et al. [14]	Sample: 50 Period: >3 months Age: 40–65 years Sex: 33 men and 17 women Losses: 0	(*n* = 25) Conventional physiotherapy + isokinetic muscle strengthening	(*n* = 25) Conventional physiotherapy	Knee and ankle Concentric flexion and extension Bilateral
Chen et al. [15]	Sample: 31 Period: <6 months Age: 51–78 years Sex: 13 men and 11 women Losses: 7	(*n* = 12) Conventional physiotherapy + isokinetic muscle strengthening	(*n* = 12) Conventional physiotherapy + isotonic muscle strengthening	Knee Concentric and eccentric flexion and extension Bilateral
Singhal et al. [16]	Sample: 30 Period: 4–21 months Age: 38–66 years Sex: 26 men and 4 women Losses: 0	(*n* = 15) Conventional physiotherapy + isokinetic muscle strengthening	(*n* = 15) Conventional physiotherapy + functional muscle strengthening	Hip, knee, and ankle Concentric flexion and extension Affected side
Gharib et al. [17]	Sample: 30 Period: 6–18 months Age: 46–62 years Sex: 16 men and 14 women Losses: 0	(*n* = 15) Conventional physiotherapy (60 min) + isokinetic muscle strengthening	(*n* = 15) Conventional physiotherapy (60 min)	Knee and ankle Concentric flexion and extension Affected side
Chang-man et al. [18]	Sample: 30 Period: >6 months Age: 42–66 years Sex: 17 men and 13 women Losses: 0	(*n* = 15) Conventional physiotherapy + isokinetic muscle strengthening in open kinetic chain (45 min)	(*n* = 15) Conventional physiotherapy + isokinetic muscle strengthening in closed kinetic chain (45 min)	Knee Concentric flexion and extension Affected side
Ran et al. [19]	Sample: 80 Period: >2 months Age: 60–75 years Sex: not provided Losses: 0	(*n* = 40) Conventional physiotherapy + 20 min aerobic exercise + 20 min isokinetic training	(*n* = 40) Conventional physiotherapy + 40 min aerobic exercise	Knee Concentric flexion and extension Affected side

**Table 3:** Type of equipment, training (dosage), and duration.

	Equipment	Training (Dosage)	Duration and Frequency
Sen et al. [14] (Turkey)	Biodex System	Warm-up: (knee) 3 reps at 60°/s and 180°/s; (ankle) 3 reps at 60°/s and 120°/s. Cool-down: not reported. Training: (knee) 5 reps at 60°/s, 90°/s, 120°/s, 150°/s, and 10 reps at 180°/s (30 total); (ankle) 5 reps at 60°/s, 90°/s, 120°/s, and 10 reps at 150°/s (25 total).	3 weeks 5 days/week (15 sessions)
Chen et al. [15] (China)	Biodex System	Warm-up and cool-down: not reported. Training: (isokinetic) 3 sets of 5 concentric + 5 eccentric reps at 60°/s (30 total); (isotonic) 3 sets of 10 reps at 60% maximum strength (30 total).	4 weeks 5 days/week (20 sessions)
Singhal et al. [16] (India)	Humac/ Norm	Warm-up and cool-down: not reported. Training: (isokinetic) 3 sets of 5 concentric reps at 60°/s (15 total per joint); (functional) step exercises (multi-directional), single-leg supports, sit-to-stand transfers, and bridges.	6 weeks 3 days/week (18 sessions)
Gharib et al. [17] (Egypt)	Biodex System	Warm-up and cool-down: not reported. Training: (knee and ankle) 3 sets of 5 concentric reps at 30°/s, and 5 eccentric at 90°/s (30 total per joint).	8 weeks 3 days/week (24 sessions)
Chang-man et al. [18] (Korea)	Biodex System	Warm-up: 5 min on a stationary bike. Cool-down: 10 min passive stretching. Training: (open and close chain) 5 sets of 5 reps at 90°/s, 8 reps at 120°/s, 10 reps at 150°/s (115 total). “Leg press”-type knee accessory used for closed-chain.	6 weeks 3 days/week (18 sessions)
Ran et al. [19] (China)	Humac/ Norm	Isokinetic warm-up: (3 min) 10 reps at 180°/s. Training: 10 reps at 180°/s, 120°/s, 90°/s and 60°/s (40 total). Aerobic warm-up: (3 min) and cool-down (2 min). Training: 15 min on a cycle-ergometer at level 12–15 on Borg scale.	4 weeks 5 days/week (20 sessions)

Reps: repetitions; s: seconds.

**Table 4:** Main findings: outcome measures, results, and conclusions.

	Muscle Strength	Gait and Mobility	Main Results and Conclusions
Sen et al. [14]	Knee PT at 60° and 180°/s Ankle PT at 60° and 120°/s	FIM SS-QOL 10-MWT 6MWT SCT TUG BBS RMI	Isokinetic training combined with physiotherapy showed significant improvements in muscle strength (*p* < 0.025), mobility, gait, balance, and quality of life, with greater results observed in the isokinetic group (*p* < 0.025, *p* < 0.05). Bilateral isokinetic training combined with physiotherapy appears to be more effective for muscle strengthening and improving functional outcomes in stroke patients.
Chen et al. [15]	Knee PT at 60° and 120°/s Isometric PT at 90°/s	SF-36 TUG	Both groups showed significant gains in knee flexion, SF-36 physical functioning, and TUG. The isokinetic group showed greater improvement, with significant differences in knee flexion at 60°/s and 120°/s (*p* < 0.05) on the affected side. If available, isokinetic training during the subacute phase of stroke may provide more significant benefits.
Singhal et al. [16]	Hip/Knee/ Ankle PT 60°/s	6MWT SL	Both groups showed significant improvements in PT 60°/s (hip and knee) and 6MWT, with the isokinetic group showing more substantial results (*p* < 0.05). While both groups improved in PT 60°/s (ankle) and SL, no significant differences were observed between them. Lower limb isokinetic training is more effective than functional training for strengthening hip and knee muscles and improving 6MWT performance.
Gharib et al. [17]	Knee/Ankle PT 30° and 90°/s	TUG Qualisys Motion Capture System	Both groups showed significant improvements in PT 30 and 90°/s. The isokinetic group showed more remarkable results (*p* < 0.05). Significant differences in gait speed, gait cycle, single-leg support, and TUG favored the isokinetic group (*p* < 0.05). Isokinetic muscle strengthening combined with physiotherapy is effective for improving muscle strength, gait, and functional mobility in chronic phase stroke patients.
Chang-man et al. [18]	Knee PT/BW 60°/s Ankle PT/BW 30°/s	GAITRite mat	Both groups showed significant improvements in PT/BW (*p* < 0.01), with no significant differences between them. Significant improvements in gait speed were seen in both groups. The closed kinetic chain exercise group showed more considerable results in gait speed and spatial symmetry (*p* < 0.01). Isokinetic strengthening in the closed kinetic chain can effectively improve sensorimotor function, gait, and functional capacity in the chronic phase of stroke rehabilitation.
Ran et al. [19]	Knee PT 60°/s TW	FMA-LE VO2 max	Both groups showed statistically significant effects on all outcome measures (*p* < 0.05). Statistically significant differences favoring isokinetic strengthening were found compared to the control group (*p* < 0.05). Isokinetic training combined with aerobic exercise is more effective for improving cardiopulmonary and lower limb function in stroke patients and can be applied in clinical practice.

PT: peak torque; FIM: Functional Independence Measure; SS-QOL: Stroke-Specific Quality of Life Scale; 10-MWT: 10-Meter Walk Test; 6MWT: 6-Minute Walk Test; SCT: Stair Climbing Test; TUG: Timed Up and Go Test; BBS: Berg Balance Scale; RMI: Rivermead Mobility Index; SF-36: SF-36 Health Survey; SL: step length; Qualisys Motion Capture System: motion capture system that evaluates gait speed, gait cycle duration, and single-limb support time; PT/BW: peak torque/body weight; GAITRite mat: portable electronic walkway that measures gait speed, step length, and swing phase duration; TW: total work; FMA-LE: Fugl-Meyer Assessment for Lower Extremity; VO_2_ max: Maximum Oxygen Uptake.

**Table 5:** Common outcome measures and results by time post-stroke (before or after 6 months).

	Time Post-Stroke	Muscle Strength	Gait and Mobility	Results
Sen et al. [14]	<6 months	Knee and ankle PT 60°/s	6MWT, TUG, RMI	Greater results in isokinetic group (*p* < 0.025, *p* < 0.05).
Chen et al. [15]	<6 months	Knee PT 60°/s	TUG	Better improvement in isokinetic group (*p* < 0.05).
Ran et al. [19]	<6 months	Knee PT 60°/s	FMA-LE	Greater results in isokinetic group (*p* < 0.05).
Singhal et al. [16]	>6 months	Hip, knee and ankle PT 60°/s	6MWT, SL	Better improvement in isokinetic group (*p* < 0.05). No differences between groups for SL and ankle PT 60°/s.
Gharib et al. [17]	>6 months	Knee and ankle PT 30° and 90°/s	TUG, gait speed	Greater results in isokinetic group (*p* < 0.05).
Chang-man et al. [18]	>6 months	Knee and ankle PT 60°/s:	Gait speed	Both groups improved muscle strength (*p* < 0.01), with no differences between them. Greater results in gait speed for CKC group (*p* < 0.01)

PT: peak torque; 6-Minute Walk Test; TUG: Timed Up and Go Test; RMI: Rivermead Mobility Index; FMA-LE: Fugl-Meyer Assessment for Lower Extremity; SL: step length; CKC: closed kinetic chain.

**Table 6:** Effect size and statistical measures.

	Effect Size or Clinical Significance	Statistical Measures
Sen et al. [14]	Not reported	*p*-value, change level
Chen et al. [15]	Not reported	*p*-value
Singhal et al. [16]	Not reported	*p*-value, percentage of improvement
Gharib et al. [17]	Not reported	*p*-value, *t*-value
Chang-man et al. [18]	Clinical significance on gait velocity: OKC 0.10 m/s, CKC 0.14 m/s.	*p*-value
Ran et al. [19]	Not reported	*p*-value, *t*-value

OKC: open kinetic chain; CKC: closed kinetic chain.

**Table 7:** Scoring of the included studies on the PEDro scale.

Author (Year)		Item										
	1	2	3	4	5	6	7	8	9	10	11	Total
Sen et al. (2015) [14]	Y	1	0	1	0	0	0	1	1	1	1	6/10
Chen et al. (2015) [15]	Y	1	1	1	0	0	0	0	0	1	1	5/10
Singhal et al. (2017) [16]	Y	1	1	0	0	0	0	1	1	1	0	5/10
Gharib et al. (2017) [17]	Y	1	1	1	0	0	0	1	1	1	1	7/10
Chang-man et al. (2019) [18]	Y	1	0	1	0	0	0	1	1	1	1	6/10
Ran et al. (2022) [19]	Y	1	0	1	0	0	1	1	1	1	1	7/10

The scoring items for the included studies on the PEDro scale are as follows: (1) the selection criteria were specified; (2) subjects were randomly assigned to groups; (3) the allocation was concealed; (4) groups were similar at baseline in relation to the most important prognostic indicators; (5) all subjects were blinded; (6) all therapists administering the therapy were blinded; (7) all evaluators measuring at least one key outcome were blinded; (8) measures of at least one key outcome were obtained from more than 85% of the subjects initially assigned to the groups; (9) results from all subjects who received treatment or were assigned to the control group were presented, or when this was not possible, data for at least one key outcome were analyzed by ‘intention to treat’; (10) statistical comparison results between groups were reported for at least one key outcome; (11) the study provides point estimates and variability measures for at least one key outcome. Note: Item 1 is not used in the calculation of the total score.

**Table 8:** Levels of evidence and grades of recommendation according to the Oxford scale.

	Levels of Evidence	Grades of Recommendation
Sen et al. [14]	1b	A
Chen et al. [15]	2b	B
Singhal et al. [16]	2b	B
Gharib et al. [17]	1b	A
Chang-man et al. [18]	1b	A
Ran L et al. [19]	1b	A

## Data Availability

All data are reflected in the document.
